# Is Sleep Essential?

**DOI:** 10.1371/journal.pbio.0060216

**Published:** 2008-08-26

**Authors:** Chiara Cirelli, Giulio Tononi

## Abstract

No current hypothesis can explain why animals need to sleep. Yet, sleep is universal, tightly regulated, and cannot be deprived without deleterious consequences. This suggests that searching for a core function of sleep, particularly at the cellular level, is still a worthwhile exercise.

Everybody knows that sleep is important, yet the function of sleep seems like the mythological phoenix: “Che vi sia ciascun lo dice, dove sia nessun lo sa” (“that there is one they all say, where it may be no one knows,” Wolfgang Amadeus Mozart and Lorenzo da Ponte [1790], *Così fan tutte*). But what if the search for an essential function of sleep is misguided? What if sleep is not required but rather a kind of extreme indolence that animals indulge in when they have no more pressing needs, such as eating or reproducing? In many circumstances sleeping may be a less dangerous choice than roaming around, wasting energy and exposing oneself to predators. Also, if sleep is just one out of a repertoire of available behaviors that is useful without being essential, it is easier to explain why sleep duration varies so much across species [1–4]. This “null hypothesis” [5–7] would explain why nobody has yet identified a core function of sleep. But how strong is the evidence supporting it? And are there counterexamples?

## Sleep Function: The Null Hypothesis

So far the null hypothesis has survived better than alternatives positing some core function for sleep [8–10]. In what follows we shall test the null hypothesis by considering three of its key corollaries. If the null hypothesis were right, we would expect to find: (1) animals that do not sleep at all; (2) animals that do not need recovery sleep when they stay awake longer; and, finally, (3) that lack of sleep occurs without serious consequences.

## Corollary 1: Are There Animals That Do Not Sleep?

Sleep is a reversible condition of reduced responsiveness usually associated with immobility. The decreased ability to react to stimuli distinguishes sleep from quiet wakefulness, while its reversibility distinguishes sleep from coma. Only a small number of species—mostly mammals and birds—have been evaluated in detail with respect to sleep. Most studies found signs of sleep, both behavioral (quiescence and hyporesponsivity) and electrophysiological (e.g., the slow waves of non-rapid eye movement [NREM] sleep). Scientists have been hesitant to attribute sleep to reptiles, amphibians, fish, and especially invertebrates, preferring the noncommittal term “rest” in the absence of electrophysiological signs resembling those of mammals and birds. Studies with Drosophila melanogaster [11,12], however, demonstrated that flies, also, become less responsive, i.e., sleep, when they remain quiescent for a few minutes. Moreover, sleep pressure increases if flies are kept awake, their sleep patterns change with the life span, and they are sensitive to hypnotics and stimulants [13–15]. Finally, the fly brain undergoes changes in gene expression between sleep and wakefulness similar to those observed in mammals [16,17], and shows changes in brain electrical activity [18]. Similar criteria have now been provided for zebrafish [19–21], and there is evidence that even the worm C. elegans shows a sleep-like state at a certain stage of development [22].

**Figure 1:**
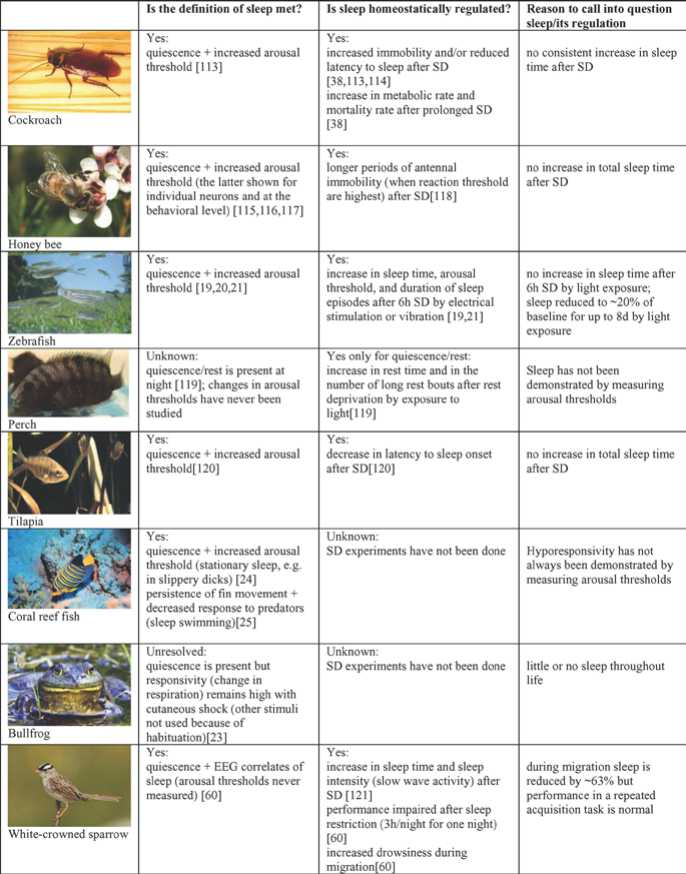
Animal Species in Which the Presence of Sleep and/or Its Homeostatic Regulation Have Been Called into Question See references [113–121]. SD, sleep deprivation.

It has been argued that the assumption that sleep is universal is based on poor evidence [7]. Figure 1 summarizes some of the “difficult” cases. The bullfrog is often promoted as an example of an animal that does not sleep. There is, however, only one study on this topic, published in 1967 [23]. This report concluded that bullfrogs do not sleep because even during the resting phase they never failed to show a change in respiratory responses after painful stimuli (cutaneous shock). The same report acknowledged that arousal thresholds could not be measured during the cyclic phases with the lowest respiratory activity, nor could they be tested with other physiological stimuli, such as light or sound. Also, the underlying assumption in that study was that shocks delivered late at night (presumably in the middle of sleep) should elicit less respiratory response than those given early in the night (when sleep had just started); however, the opposite was found [23]. In fact, we now know that in rodents and humans the deepest sleep occurs early after sleep onset. At the very least, it seems that more experiments are needed before concluding that bullfrogs do not sleep.

Coral reef teleosts showing sleep swimming have similarly been used as evidence that not all animals sleep (Figure 1). Two types of reef fish have been studied in terms of sleep; one is immobile at night and less responsive to alerting stimuli (stationary sleep [24]), and another [25] retreats to the coral at night, where it continues to move its fins even when holding a fixed position (called “sleep swimming”; possibly to avoid hypoxia [25]). The researchers who studied these teleosts defined sleep swimming as a state “equivalent to sleep.” They assumed that sensory information must still be processed to a certain extent during sleep swimming, because each individual remains in its swimming zone during the night. Yet, the fish at night loses the ability to respond to predators [25], and mortality due to predators' attacks is much higher at night, when the fish is sheltering in corals, than during the day, when it feeds in open waters [26]. Most losses to predators occur in the first 1–2 h after sunset, i.e., at the beginning of the “rest” period. Although limited, the available evidence seems to suggest that sleep swimming is associated with hyporesponsivity.

**Figure 2:**
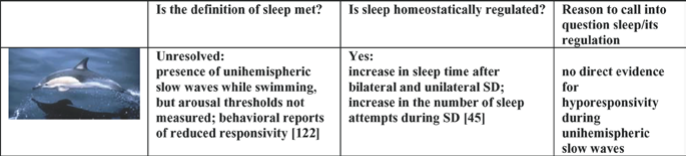
Sleep in Dolphins: A Difficult Case? Dolphins and a few other species have developed unihemispheric (one-sided) sleep, a remarkable specialization strongly suggesting that sleep must have some essential function and cannot be eliminated [123]. Yet, the very presence of sleep in dolphins has recently been questioned based on four lines of evidence. (1) It has been argued that unihemispheric sleep is not really sleep because dolphins move and, more crucially, because there is no evidence that the hemisphere with slow waves is less capable of responding to the environment [7]. In fact, the presence of slow waves in one hemisphere is associated with unilateral slow waves in the thalamus, and unilateral decrease in brain metabolism, including in the ipsilateral locus coeruleus, an arousal-promoting system [124]. In other words, electrophysiological and metabolic processes that always occur bilaterally in the brain of other mammals can be engaged unilaterally in the brain of a dolphin. It therefore seems at least plausible that half of the dolphin brain can be unresponsive while the other half may be awake. Indeed, there are a few brief reports showing that both bottle-nosed and white-sided dolphins show reduced or no response to stimuli when performing stereotypical circular swimming, which is associated with unihemispheric sleep [122,125]. (2) Another argument was raised by a report describing continuous activity in newborn dolphins (and whales) and their mothers for the first postpartum month [126]. However, based on 2 additional recent studies that assessed eye closure under water [127,128], it seems likely that young cetaceans engage in unihemispheric sleep while swimming, and do so from birth for many hours a day. It has been argued [7] that even if present, this unihemispheric sleep could not be restorative, because it is interrupted every 30–60 s by breathing. The basis for the latter assumption remains unclear. Rats sleep 12–14 h per day and their sleep cycle lasts approximately 10–20 min. When forced to a schedule of 30-s stimulation on/90-s stimulation off, rats learn quickly to sleep in the 90-s off period, so that their total daily time in NREM sleep does not change (and the intensity increases [61]). When tested in a spatial learning task, these animals, which are not capable of unihemispheric sleep, still perform at 70%–80% of baseline levels. Until (if ever) combined EEG-performance studies will be performed in young cetaceans, it seems at the very least premature to imply that their sleep must necessarily be poor and not restorative. Even so, the issue is not how well they sleep, but whether they sleep. (3) Regarding sleep homeostasis, there is only one publication [45] in which EEG recordings were used in dolphins to measure the response to sleep deprivation. Of note, the study used different lengths of sleep deprivation (35–150 h) and of recovery (9–24 h), slow waves could not be totally prevented during the sleep deprivation, recovery ended at different time of day, data were averaged for the entire recovery period, and only sleep duration (not sleep intensity) was measured. Nevertheless, it was found that (i) during sleep deprivation the amount of stimulation required to prevent slow waves increased progressively; (ii) in all cases (*n* = 6) bilateral sleep deprivation increased sleep time during recovery; (iii) in all cases (*n* = 9) unihemispheric sleep deprivation increased sleep duration in the affected hemisphere. Though the results of this seminal study have been characterized as “very variable” [7], it is hard to deny the presence of a clear-cut sleep rebound. (4) A final issue was raised by a recent study in two highly trained dolphins, which showed that they could maintain continuous vigilance for 5 d [125]. In fact, during the stimulation period the two animals displayed resting behavior at night (floating or very slow stereotyped swimming), and response times were slower at night than during the day, suggesting that at least some rest was obtained (most likely unihemispheric sleep, as suggested by the authors). Moreover, one of the two dolphins the last night “ignored all target stimuli for 4 hours and appeared to be asleep” [125].

In dolphins the very presence of sleep has been called into question because these marine mammals move continuously and their arousal thresholds have not been measured directly (Figure 2). Yet, dolphins are capable of engaging in slow waves with half of the brain at a time, a property called “unihemispheric sleep” [27–31]. Moreover, there is some limited evidence of decreased response to stimuli during stereotypical circular swimming, which is associated with unihemispheric sleep (Figure 2). The very fact that dolphins have developed the remarkable specialization that is unihemispheric sleep, rather than merely getting rid of sleep altogether, should count as evidence that sleep must serve some essential function and cannot be eliminated. Thus, there is no clear evidence of a species that does not sleep.

## Corollary 2: Can Sleep Loss Occur without a Compensatory Rebound?

Are there animals in which sleep is not homeostatically regulated? Cockroaches, honeybees, and tilapia (Figure 1) are seen as species lacking this mechanism, because their response to sleep deprivation does not consistently include an increase in sleep time. However, it is well known that sleep has both a quantitative (duration) and a qualitative (intensity) dimension [32,33]. Sleep can be recovered by sleeping longer, more deeply (for instance in mammals NREM sleep becomes richer in slow waves), and/or in a more consolidated manner (sleep is less frequently interrupted by brief awakenings). Claims that in some animals sleep is not homeostatically regulated should be made only after several aspects of the response to sleep loss have been analyzed, including changes in sleep intensity and pattern.

Evidence of apparent lack of sleep rebound comes from an early study of sleep deprivation using constant light in the pigeon [34], in which sleep was nearly eliminated in the birds for more than 10 d, with no subsequent increases in either total sleep time or slow-wave activity (SWA). Considered one of the best markers of sleep intensity, SWA is a measure of the number and amplitude of slow waves during NREM sleep [35]. However, in this study the overall amount of SWA was preserved across the entire sleep deprivation period in constant light, suggesting that the increasing sleep pressure may have forced sleep slow waves to leak into wakefulness.

There is evidence that zebrafish sleep and show sleep rebound after sleep is prevented by electrical or mechanical stimulation but not by light exposure, which can drastically reduce sleep for several days [19–21]. We interpret these findings to mean that light is a powerful arousing stimulus in zebrafish, not that sleep in this animal is dispensable. Even with light exposure, 15%–20% of baseline sleep remains, and this percentage increases if constant light is maintained for more than one week [21]. Moreover, it is unknown whether in zebrafish prolonged light exposure affects sleep intensity or causes long-term detrimental effects.

In the dolphin, not only the existence of sleep itself, but sleep homeostasis has been questioned also. The single published study on this issue, however, clearly shows that unihemispheric sleep is homeostatically regulated (Figure 2).

By reviewing the data used to support the claim that sleep is not universal [7], we instead reach the opposite conclusion: sleep is present and strictly regulated in all animal species that have been carefully studied so far.

## Corollary 3: Can Sleep Loss Occur without Negative Consequences?

Harmful consequences of sleep deprivation have been described in many studies. Most dramatically, prolonged sleep deprivation leads to death. Rats kept awake using the disk-over-water method develop a peripheral syndrome characterized by increased metabolic rate and decreased body weight, which culminates in death after 2–4 wk [36]. Prolonged sleep deprivation is also fatal in flies [37], cockroaches [38], and humans with fatal familial insomnia, who die after developing a syndrome not unlike that seen in sleep-deprived rats [39]. Pigeons, however, appear capable of surviving prolonged sleep deprivation [40]. Prolonged sleep deprivation has not been studied in other species. Thus, it is unclear whether death, when it occurs, is due to loss of sleep per se or to other factors, such as forced arousals and the associated stress.

### Sleep intrusion.

Whether or not sleep loss is lethal, sleep deprivation has two consequences that never fail to occur (but see Figure 2). The first one is intrusion of sleep into wakefulness. When wakefulness is enforced, sleep pressure increases and sleep cannot be avoided, irrespective of stimulation. During short-term (6–24 h) sleep deprivation experiments, some portion of baseline sleep (usually 5%–10%) is always maintained (e.g., flies [15], zebrafish [21], mice [41], rats [42], rabbits [43], hamsters [44], and dolphins [45]). Under a chronic “total” sleep deprivation regimen, rats still sleep at least 10% of the time, due to “microsleep” episodes [36]. Perhaps even more important, spectral analysis of the electroencephalogram (EEG) reveals that slower EEG activity (delta, < 4 Hz; or theta, 4–7 Hz) leaks into periods during which the animal may be moving around with eyes open, and which are therefore conventionally scored as wakefulness [42,46].

It is easier to keep humans awake. Especially motivated subjects can be kept awake for up to several days (for 11 d in the famous case of Randy Gardner [47]) by keeping busy with pleasurable activities. (Although seriously sleep deprived humans have been reported to fall asleep even in the most dangerous situations [48].) People may seem superficially awake (moving and with eyes open) even though the EEG slows down or exhibits microsleeps [49,50]. Few studies so far have investigated the leakage of slower brain activity in the EEG of sleep deprived humans, though several studies show an increase in power in the theta frequency bands with prolonged wakefulness and sleep deprivation [50,51].

It is unknown whether the presence of slower activity in the “wake” EEG spectra of sleep-deprived animals or humans is due to “piecemeal” sleep, where some brain regions may be asleep whereas others are awake [52], to “salt and pepper” sleep-wake, in which within the same brain regions individual neurons may be awake (depolarized) and others may be oscillating between up- and downstates (asleep, [53]), or to abnormal cellular activity that is neither wake or sleep. Whatever the underlying cellular events, it seems impossible to completely deprive an animal of sleep for more than 24 h [54]. Rather, what seems to occur is a kind of “dormiveglia” (sleepwake), a mixed state that is clearly dysfunctional.

### Cognitive impairment.

The second documented consequence of sleep deprivation is performance deterioration, especially cognitive impairment. Intriguingly, there is great inter-individual variability in the susceptibility of humans to the effects of sleep deprivation, and subjects whose performance is little impaired by one task may show great impairment in another task [55,56]. Partial sleep restriction also impairs cognitive performance, although subjects may not realize that they are impaired [57,58]. Cognitive impairment is easier to study in humans than in animals, but there is now evidence that both acute sleep loss and sleep restriction affect cognitive function in flies [59], birds [60], and rodents (e.g., [61]).

### Sleepy or tired?

An important unsolved question is whether the impairment, cognitive or otherwise, that follows sleep deprivation is merely the consequence of an increased drive for sleep (“sleepiness”) or whether brain cells need sleep because they are actually “tired.” Pure sleepiness can be conceptualized as the effect of central sleep-promoting mechanisms telling the brain it is time to sleep, whether or not brain cells need to do so. For instance, when we are jet-lagged, the circadian system may at times dampen the activity of arousal systems and boost that of sleep-promoting systems in brainstem, hypothalamus, and basal forebrain [62], even though we may not have been awake for long and presumably do not need extra sleep. Attention lapses or unresponsiveness in such circumstances could be due to the activation of sleep-promoting mechanisms, not to the brain being actually “tired.” Similar considerations apply to the increased sleepiness that follows a heavy meal, the use of sedatives, a boring environment, and so on.

Conversely, it may be that brain cells actually do get tired as a function of waking activities, whether or not the arousal systems are pushing the organism to stay awake. This may be the case, for instance, when we try to prolong wakefulness using amphetamines or other arousal-promoting drugs: though we are alert, certain aspects of performance seem to deteriorate [63]. Pure tiredness can be conceptualized as the inability of brain cells to continue functioning in their normal waking mode, despite the central wake-promoting mechanisms telling the brain it should be fully alert. PET studies show that glucose metabolism decreases more in prefrontal and parietal association areas involved in attention, judgment, and associative functions than in primary sensory and motor areas [64–67]. These results are more consistent with some parts of the brain being disproportionately “tired” than with the entire brain being “sleepy.”

Altogether, then, while we still do not understand whether sleep deprivation is followed by sleep intrusions and cognitive impairment because we become sleepy, tired, or both, the evidence so far indicates that, contrary to the predictions of the null hypothesis, lack of sleep has serious consequences, especially for the brain.

## Sleep Function: Beyond the Null Hypothesis

The three corollaries of the null hypothesis do not seem to square well with the available evidence: there is no convincing case of a species that does not sleep, no clear instance of an animal that forgoes sleep without some compensatory mechanism, and no indication that one can truly go without sleep without paying a high price. What many concluded long ago still seems to hold: the case is strong for sleep serving one or more essential functions [9,10]. But which ones? The points below represent judgment calls that may be helpful in provoking discussions, guiding hypotheses and, above all, inspiring experimental tests.

### A universal function.

It may still be wise to search for a function or functions that apply to all animals. It is unknown whether a proto-sleep state emerged early in evolution, perhaps out of the rest–activity cycle, or whether sleep emerged multiple times in the course of evolution. In either case, the simplest hypothesis (after the null hypothesis) is that sleep evolved to serve the same function in all species.

### A core function.

There is no doubt that sleep, by changing so many aspects of physiology and behavior, affects the vast majority of body functions, from immunity to hormonal regulation to metabolism to thermoregulation. However, the simplest hypothesis (after the null hypothesis) is that there may be a single core function that requires sleep, and adventitious functions that take advantage of sleep.

### A function transcending specific phenotypes and mechanisms.

Sleep comes in many forms. In the best known example, brain activity in NREM sleep and REM sleep is remarkably different: the EEG of NREM sleep is distinctive, with slow waves and spindles, and the EEG of REM is similar to that of wakefulness [68]. Brain metabolism is low in NREM sleep but high in REM sleep [69]. Thermoregulation is preserved in NREM sleep but not in REM sleep [70]. It is therefore assumed that these two phases of sleep perform quite different functions. It is highly unlikely that fly brains can produce slow waves or spindles [18], and they do not seem to have the equivalent of REM sleep. The mechanisms of sleep can also vary considerably: the hypocretin–orexin system has an arousing action in mammals but may have a hypnogenic effect in zebrafish [21]. It may be, of course, that each variation in sleep phenotype or mechanism implies a different function (and to some extent functional differences must exist), but it is perhaps more parsimonious to assume that there may be many ways to achieve the same goal. After all, in NREM as in REM stages, in fruit flies as in zebrafish as in humans, the organism (or parts of it) is quiescent and unresponsive—that is, asleep.

### A neural function.

Although the entire body benefits from sleep [71], the most immediate, unavoidable effect of sleep deprivation is cognitive impairment. The brain suffers most from sleep deprivation. It is less clear that the rest of the body suffers as rapidly, significantly, or inevitably from lack of sleep. Although we talk about a muscle that is active or at rest, muscle rest can be achieved during quiet wakefulness, and does not seem to require sleep. However, few studies have compared directly the restorative value of quiet wakefulness and sleep for either the brain or any other organ [48,72]. This is a research approach that clearly deserves more emphasis in the future.

### A cellular function.

If sleep has a core function involving the brain, such a function might be identifiable at the cellular level and there would be a price for brain cells to remain indefinitely awake. Indeed, the search for the function of sleep has often focused on identifying neuronal resources depleted during wakefulness and restored during sleep or, alternatively, neurotoxic substances that accumulate during wakefulness and dissipate during sleep. In mice, sleep may favor the replenishment of glycogen in glial stores [73], but this may be the case in only a few brain regions, and not in all mouse strains [74,75]. It has also been proposed that sleep may allow the removal of toxic free radicals accumulated in the brain during wakefulness [76,77]. However, studies in long-term sleep deprived rats found evidence for oxidative stress, but not oxidative damage (e.g., [78,79]). This result suggests that the cellular stress response induced during wakefulness may be sufficient to avoid long-term negative effects [80,81]. Other possibilities that are worth exploring are inspired by the recent systematic data on changes in brain gene expression that occur between sleep and wakefulness or after sleep deprivation [16,17,80,82–89]. In all species studied (flies, mice, rats, hamsters, and sparrows), wakefulness leads to the up-regulation of three categories of transcripts—those involved in energy metabolism, in the response to cellular stress, and in activity-dependent processes of synaptic potentiation. By contrast, transcripts expressed at higher levels during sleep are involved in synaptic depression and depotentiation, in the synthesis/maintenance of membranes, and in lipid metabolism [80,87]. One way to make sense of these apparently disparate findings is in terms of plastic processes. For example, we have suggested that during wakefulness, when animals interact with the environment and need to learn, there is a net increase in synaptic strength in many brain areas, in which case sleep would be needed to renormalize such changes [90,91]. A net increase of synaptic strength at the end of a waking day would result in higher energy consumption [92,93], larger synapses that take up precious space [94], and saturation of the capacity to learn. Also, a net strengthening of synapses likely represents a major source of cellular stress [80–82], due to the need to synthesize and deliver cellular constituents ranging from mitochondria to synaptic vesicles to various proteins and lipids. In this view, then, sleep would be necessary to renormalize synapses to a baseline level that is sustainable and ensures cellular homeostasis.

### A function that cannot be provided by quiet wakefulness and that benefits from environmental disconnection.

If wakefulness were as good as sleep in fulfilling a fundamental biological function (or even nearly as good), is it likely that sleep would be so ubiquitous? Why would an animal choose to spend long periods of time not just immobile, but above all disconnected from the environment? It would seem that, if sleep has a core function, and if this function is for the brain, it should be one the brain cannot fulfill during wakefulness, and one that benefits from being performed off-line. Among several options, those related to plasticity and memory are especially intriguing, not least since during sleep, despite the functional disconnection from the environment, most neurons remain spontaneously active at levels similar to wakefulness [95].

Off-line activity may be necessary to stimulate synapses that remain underused during the waking day [96–98], so they can be ready when their turn comes. It may also be an excellent way of maintaining old memories by keeping them “exercised,” or of weakening nonadaptive memory traces while strengthening the adaptive ones [99]. A related idea is that an offline activation of neural circuits may be especially important during development [100], perhaps to rehearse innate behavioral patterns [101]. And perhaps sleep may even favor the formation of new synaptic contacts to refresh the repertoire of circuits available for the selection and acquisition of new memories [102].

Alternatively, sleep may be a good time for consolidating and integrating new memories without interference from ongoing activities, and indeed human studies have provided evidence for sleep-dependent memory consolidation, at least in some tasks [103,104]. Consolidation may happen, for instance, by further strengthening synapses already potentiated during wakefulness [103,105,106]. The observation that neural circuits activated during learning are “reactivated” during sleep is consistent with this possibility (e.g., [107–111]). Another possibility is that signal-to-noise ratios may increase through the generalized downscaling of synapses, as synapses mediating firing patterns predictive of postsynaptic activation would “survive” better than random ones [90,91,112]. This scenario would prevent runaway synaptic potentiation and the saturation of the ability to learn. Moreover, it would dovetail nicely with the cellular need for synaptic homeostasis: renormalizing synapses during sleep would counteract the cellular stress brought about by synaptic potentiation during wakefulness.

## Conclusion

While there is still no consensus on why animals need to sleep, it would seem that searching for a core function of sleep, particularly at the cellular level, remains a worthwhile exercise. Especially if, as argued here, sleep is universal, tightly regulated, and cannot be eliminated without deleterious consequences. In the end, the burden of proof rests with those who are attempting not only to reject the null hypothesis, but to gather positive evidence for the elusive phoenix of sleep.

## Abbreviations

EEG: electroencephalogram
NREM: non-rapid eye movement
SWA: slow-wave activity
